# A novel laboratory method to simulate climatic stress with successful application to experiments with medically relevant ticks

**DOI:** 10.1371/journal.pone.0275314

**Published:** 2022-09-29

**Authors:** Caleb Nielebeck, Sang Hyo Kim, Lauren Dedmon, Mark Pangilinan, Jahred Quan, William Ota, Javier D. Monzón

**Affiliations:** Natural Science Division, Pepperdine University, Malibu, California, United States of America; University of Kentucky College of Medicine, UNITED STATES

## Abstract

Ticks are the most important vectors of zoonotic disease-causing pathogens in North America and Europe. Many tick species are expanding their geographic range. Although correlational evidence suggests that climate change is driving the range expansion of ticks, experimental evidence is necessary to develop a mechanistic understanding of ticks’ response to a range of climatic conditions. Previous experiments used simulated microclimates, but these protocols require hazardous salts or expensive laboratory equipment to manipulate humidity. We developed a novel, safe, stable, convenient, and economical method to isolate individual ticks and manipulate their microclimates. The protocol involves placing individual ticks in plastic tubes, and placing six tubes along with a commercial two-way humidity control pack in an airtight container. We successfully used this method to investigate how humidity affects survival and host-seeking (questing) behavior of three tick species: the lone star tick (*Amblyomma americanum*), American dog tick (*Dermacentor variabilis*), and black-legged tick (*Ixodes scapularis*). We placed 72 adult females of each species individually into plastic tubes and separated them into three experimental relative humidity (RH) treatments representing distinct climates: 32% RH, 58% RH, and 84% RH. We assessed the survival and questing behavior of each tick for 30 days. In all three species, survivorship significantly declined in drier conditions. Questing height was negatively associated with RH in *Amblyomma*, positively associated with RH in *Dermacentor*, and not associated with RH in *Ixodes*. The frequency of questing behavior increased significantly with drier conditions for *Dermacentor* but not for *Amblyomma* or *Ixodes*. This report demonstrates an effective method for assessing the viability and host-seeking behavior of tick vectors of zoonotic diseases under different climatic conditions.

## Introduction

Ticks transmit many viral, bacterial, and protozoan pathogens to humans, domesticated animals, and wildlife, with novel emerging tick-borne diseases being identified regularly [1–3]. Worldwide, ticks are second to mosquitoes as the most prevalent vector of zoonotic disease-causing pathogens of humans [4]. However, ticks are the most common cause for vector-borne diseases of humans in North America and Europe [5, 6]. In particular, the lone star tick (*Amblyomma americanum*), American dog tick (*Dermacentor variabilis*), and black-legged tick (*Ixodes scapularis*) are the most medically relevant ticks in the United States [7]. The lone star tick is a major vector of several pathogens that cause diseases such as ehrlichiosis, southern tick-associated rash illness, and tularemia. The American dog tick is the primary vector of the bacterium that causes Rocky Mountain spotted fever. The black-legged tick is the main vector of the bacterium that causes Lyme disease, the most common tick-borne disease in the United States.

The geographic distributions of the lone star tick, American dog tick, and black-legged tick have expanded in recent decades [7, 8]. The range expansions of these ticks have been largely attributed to climate change [9, 10] and are shifting zoonotic disease risks throughout the United States and Canada [11–15]. For example, warmer and wetter winters or hotter and drier summers affect rates of survival in ticks [16–20]. Many species in *Amblyomma*, *Dermacentor*, *Ixodes* and other genera are expanding or expected to expand their ranges [21], making studies of tick ecology increasingly pertinent.

Although correlational evidence suggests that climate change is driving the range expansion of ticks, it is important to develop a mechanistic understanding of how climatic factors affect the survival, physiology, and behavior of tick vectors. Various laboratory experiments have long established, as far back as the 1940s, the importance of relative humidity (RH) on the biology of *A*. *americanum* [22–24], *D*. *variabilis* [25, 26], and *I*. *scapularis* [27–29]. Ticks generally do not drink water, so they rely on absorbing water vapor from humid air to maintain water balance while off-host, which is >90% of their life [30, 31]. This strong effect of RH on tick physiology at all life stages limits their geographic distribution and influences their behavior. For example, questing, an ecologically important host-seeking behavior in which ticks climb up vegetation and adopt a sit-and-wait strategy with extended forelegs to latch onto a passing host [32], is affected by climate and weather conditions [29, 33–35].

One limitation of experimentally manipulating RH in the laboratory is the difficulty of the actual setup. Researchers typically use saturated or calibrated salt solutions to create a specific RH environment. This method requires a stock of laboratory-grade chemicals, such as ammonium sulfate, calcium nitrate, ammonium phosphate, sodium nitrate, and potassium sulfate. Some of these salts and their solutions are corrosive and present health and environmental hazards if swallowed, inhaled, spilled, or discarded improperly. An alternative to salt solutions is a digital climate chamber or incubator that can reliably maintain a set RH, but laboratory incubators with RH functionality are very expensive. Another limitation of previous studies pertains to how ticks are contained. Researchers typically place many ticks together in a jar or vial; for example, Rodgers et al. placed 20 ticks per vial to investigate how duration of exposure to low humidity affects survival of *I*. *scapularis* [28]. Although larval ticks form aggregations under climatically stressful conditions to conserve water and promote survival [36, 37], aggregating behavior is not typically observed in nymphal or adult ticks in nature. This raises concerns about pseudoreplication in experiments that do not examine ticks individually; therefore, it remains important to avoid pseudoreplication with rigorous experimental designs that isolate individual adult ticks. We developed a novel, safe, and economical protocol for manipulating the temperature and RH microclimate of individual ticks to investigate their physiology, behavior, and survival. The purpose of this study was to test our experimental protocol and investigate how humidity influences the survival and questing behavior of the three most medically relevant species of ticks in the United States.

## Materials and methods

The protocol described in this peer-reviewed article is published on protocols.io, dx.doi.org/10.17504/protocols.io.rm7vzyo8rlx1/v4 and is included for printing as S1 Appendix with this article.

### Validation of protocol

To demonstrate that the protocol achieves its intended purpose and is an improvement over the classical method of using saturated salt solutions, we compared the cost, preparation time, and sorption performance of three humidity control packs and three inorganic salts. We used the 32%, 58%, and 84% 8-gram packs from Boveda Inc. and magnesium chloride (MgCl_2_), calcium nitrate (Ca(NO_3_)_2_), and potassium chloride (KCl) from Fisher Scientific Inc. These three hygroscopic salts have been used since the 1960s in experiments of arthropod physiology to set a fixed RH [38]. We placed either a single 8-gram pack or 100 mL of a saturated solution in a 2-L airtight container, and we placed all six containers in a laboratory incubator. We monitored the temperature and RH every minute with HOBO data loggers. In an initial 30-hour trial at a constant temperature of 25°C, we evaluated four sorption performance metrics: (1) RH set rate, defined as the time to halfway between ambient room RH and the equilibrium RH; (2) accuracy, defined as the absolute value difference between the target RH and mean RH recorded from 18 to 24 hours after setup; (3) stability, defined as the standard deviation of RH recorded between 18 to 24 hours after setup; (4) resilience, defined as the time to halfway between disturbed RH and equilibrium RH after the airtight containers were opened for 1 minute at 24 and 27 hours. Additionally, after the humidity control packs and salt solutions reached equilibrium RH, we conducted a second 24-hour trial in which the incubator temperature cycled between 20°C and 30°C, changing by 2.5° every three hours. In this second trial, we evaluated stability, defined as the standard deviation of RH recorded between 0 to 24 hours after reaching equilibrium. Lower values of all these metrics indicate a better sorption performance.

### Experimental setup

**Fig 1:**
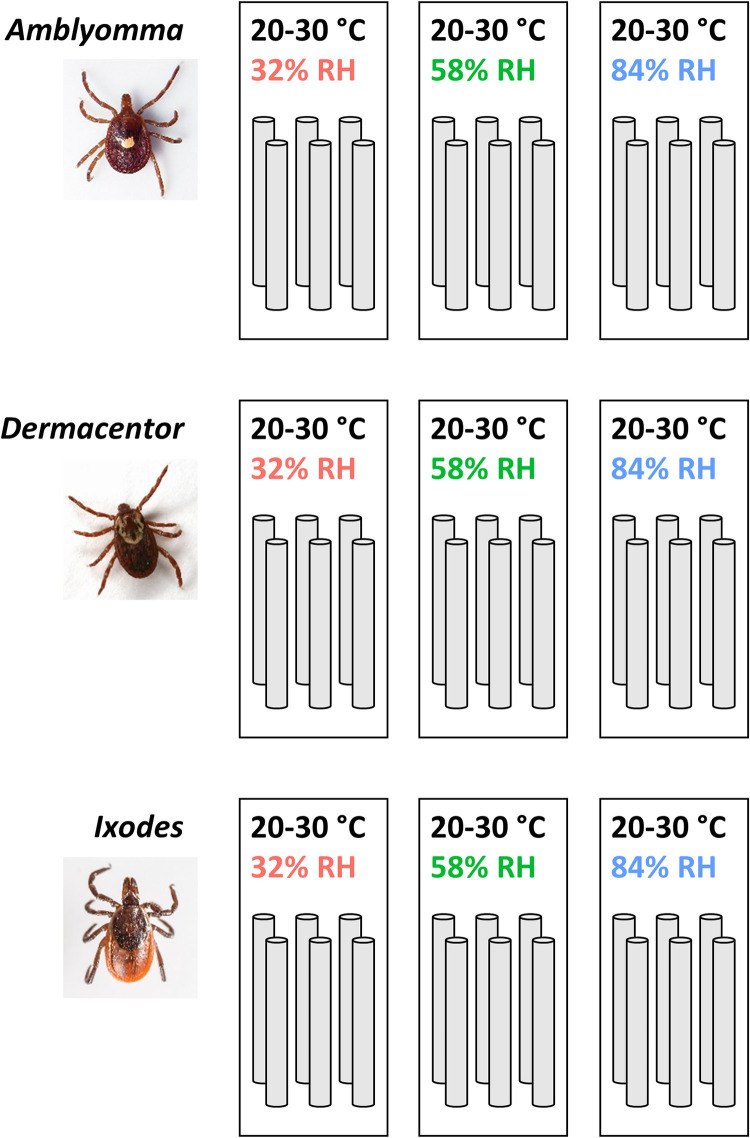
The experimental setup. Each cylinder represents a plastic tube containing one tick, and each rectangle represents an airtight container with a constant RH maintained by a humidity control pack. There were nine distinct groups based on species and RH. Each container with six ticks was replicated four times for a total sample of 24 ticks per group.

We used 216 adult female ticks from three different species: 72 *Amblyomma americanum*, 72 *Dermacentor variabilis*, and 72 *Ixodes scapularis*, and placed them into individual plastic tubes (20 cm height x 2.5 cm diameter). In each tube, we placed a 20-cm wooden skewer to provide a perching surface for the ticks in addition to the tube’s plastic surface. We secured the opening of the tubes with either a mesh screen fastened with a rubber band or the tube’s cap with drilled holes to permit free circulation of air. We divided the 216 tubes into 36 2-L airtight containers– 12 containers for each species and six tubes per container. For each species, we separated the 12 containers into three groups of four and placed a two-way humidity control pack into each airtight container to create three climate groups: 32%, 58%, and 84% RH. Thus, each container with six ticks was replicated four times for a total of 24 ticks per group and nine total groups (Fig 1). The humidity control packs use a combination of salts and water to set and maintain a predetermined RH inside the containers. We positioned the 12 containers at random into their own designated plastic tray, which we then placed onto one of the three racks of an incubator at random. We programmed the incubator to cycle between 20°C and 30°C in a 24-hour period every day, changing by 2.5° every three hours. We also programmed the incubator with a 12:12 photoperiod to turn the lights on from 9 AM to 9 PM and off from 9 PM to 9 AM. We used a HOBO data logger to independently record temperature and RH inside the containers in hourly intervals.

### Data collection

For 30 consecutive days, we checked the ticks daily from 9 AM to 12 PM to collect data during the warmest period in the daily cycle (30°C). We observed each tick daily for approximately 1 to 5 minutes. Specifically, we recorded (1) the number of ticks questing, which we defined as the tick remaining still with its front legs extended and elevated off the surface, (2) the questing height of all questing ticks rounded to the nearest 0.5 cm, and (3) the survivorship of the ticks in each of the nine groups. When a tick appeared dead, we exhaled into the tube because ticks are attracted to the carbon dioxide of a potential host and become responsive at the prospect of obtaining a blood meal. If a tick failed to respond after 5 minutes, we considered it dead and disposed of the tick in 70% ethanol. After checking all ticks, we returned the containers to their designated species tray in a random position and then returned each tray onto one of the three racks of the incubator at random. Therefore, the spatial position of each tick inside the incubator was random each day.

### Statistical analyses

To elucidate the relationship between RH and survivorship, we used Akaike’s Information Criterion (corrected for small sample sizes, AIC_c_) to evaluate and select the most plausible of the following four linear models: RH alone, species alone, RH and species combined, and RH and species with interaction effects. The response variable in all models was “day of death.” To evaluate the strength and appropriateness of each model, we used the Akaike weight (AIC_wt_), which is the probability that a model is the most parsimonious among those considered. We analyzed the survivorship data using Kaplan-Meier survival probability estimates and pairwise log-rank analysis to compare survival among the nine groups. Specifically, we compared the three RH groups within the same species to determine if there were intraspecific differences in the way individuals respond to different RH treatments. We also compared groups across species within the same RH treatment to determine if there were interspecific differences in the way species respond to the same humidity. We used the Holm-Bonferroni method to account for multiple comparisons and ensure a family-wise error rate ≤ 0.05.

We conducted Kruskal-Wallis one-way analyses of variance to compare questing height and questing frequency among groups. We only compared groups within the same species to determine if humidity affects questing behavior. Since questing frequency is a group-level proportion, we omitted data collected after the survivorship dropped below 33% (i.e., less than eight ticks in the group remained alive) to minimize the disproportionate effect that one or a few ticks in a small group would have on questing frequency.

## Results

Our method of manipulating the internal climate of individual containers with humidity control packs was substantially less costly and required minimal preparation time, compared to the classical method of using saturated salt solutions (S2 Appendix). Additionally, our method is faster at achieving equilibrium RH, more accurate at achieving the target RH, more resilient in returning to equilibrium after being disturbed, and more stable in constant and cycling temperatures (S2 Appendix).

**Fig 2:**
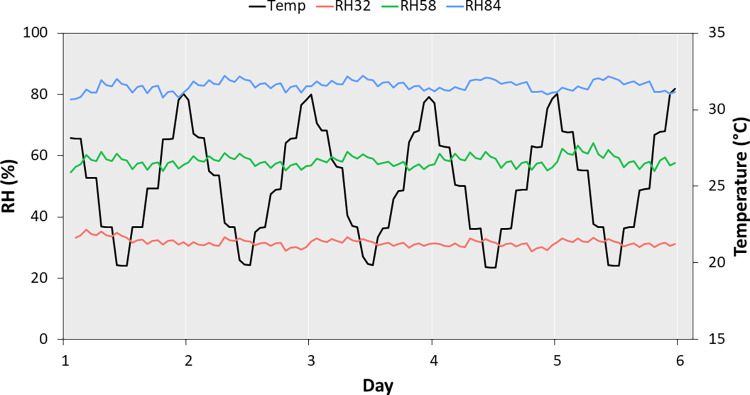
Confirmation of experimental climates. A HOBO Data Logger recorded temperature and RH hourly, confirming our setup worked as intended. The plot shows data for six days with temperature cycling between 20°C and 30°C, and RH remaining constant inside each container at approximately 32%, 58%, or 84%.

Beyond the 30-hour and 24-hour validation trials, the protocol was stable and effective for longer experiments on ticks, as independently confirmed by 118 hourly records (Fig 2). The average temperature was 25.2°C (SD = 3.4, range = 19.7–31.4). The 32% RH packs achieved a mean RH of 31.7% (SD = 1.3, range = 28.8–35.8). The 58% RH packs achieved a mean RH of 58.3% (SD = 1.8, range = 54.9–64.2). The 84% RH packs achieved a mean RH of 82.9% (SD = 1.7, range = 78.7–86.1). Importantly, the target RH was reset very quickly, in a matter of minutes, after briefly opening the containers, which was necessary to check on the ticks that appeared dead.

**Fig 3:**
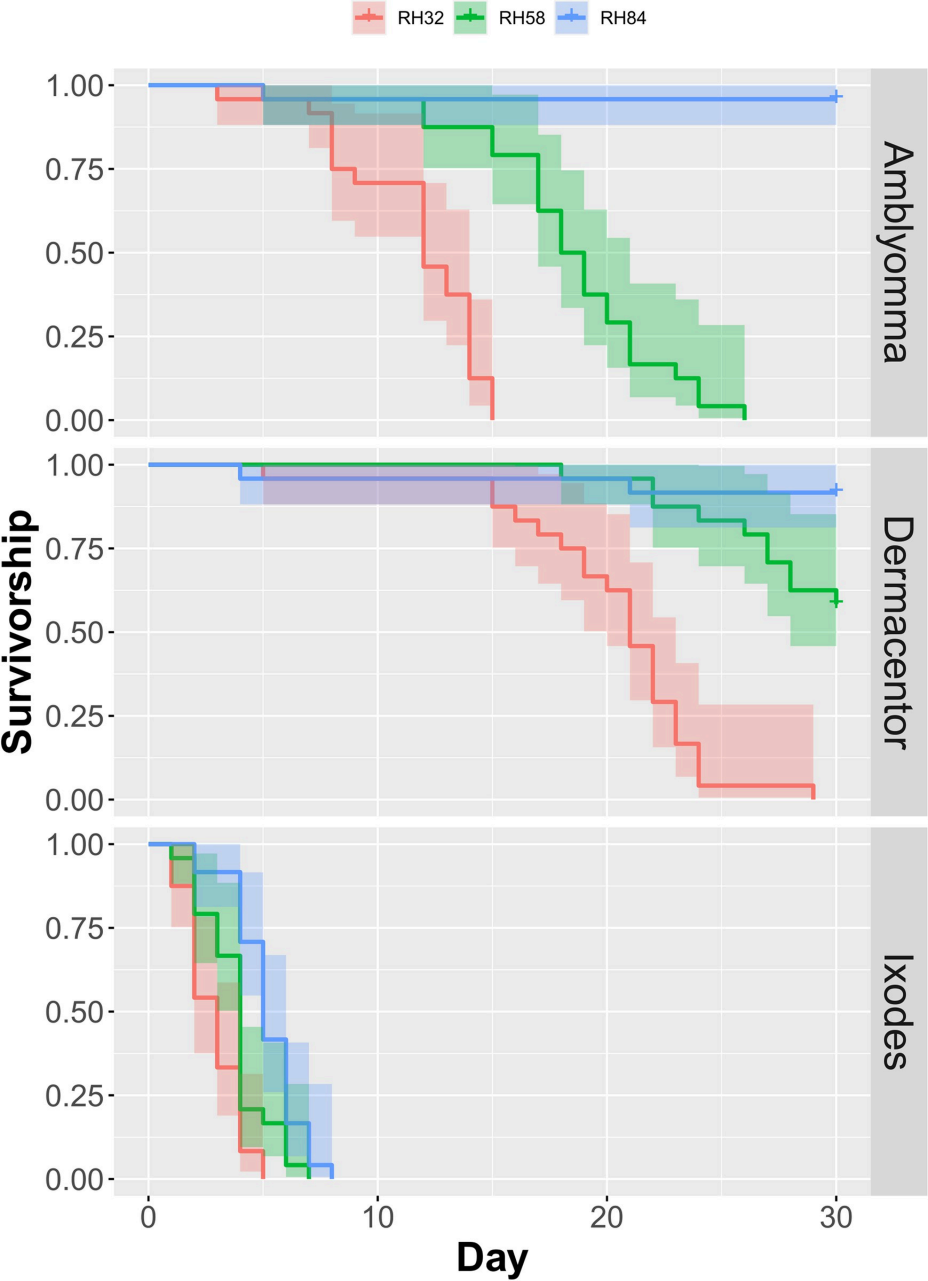
Survivorship of ticks in nine experimental groups. All within-species comparisons, and all between-species comparisons at the same RH, showed significant differences in survivorship (P < 0.05), except between *Amblyomma* and *Dermacentor* at RH84 (P = 0.55).

In all three species, survivorship was positively associated with RH, with ticks dying faster in drier conditions (Fig 3). However, there were important differences among species. Assuming an upper limit of 30 days (the total duration of the experiment), restricted mean survival times in *Amblyomma* ticks were 11.6 days, 18.3 days, and 29.0 days at 32%, 58%, and 84% RH, respectively. In *Dermacentor* ticks, restricted mean survival times were 20.3 days, 28.0 days, and 28.5 days at 32%, 58%, and 84% RH, respectively. In *Ixodes* ticks, restricted mean survival times were 2.8 days, 3.8 days, and 5.2 days at 32%, 58%, and 84% RH, respectively. Since the linear model that best explains the patterns of survivorship includes interaction effects between species and RH (Table 1), we include all within-species comparisons and all between-species comparisons at the same RH; all of which showed significant differences in survivorship, except between *Amblyomma* and *Dermacentor* at 84% RH (P = 0.55).

**Table 1:** AIC analysis of general linear models describing the influence of species and relative humidity on tick survivorship.

Model type	K	ΔAIC_c_	AIC_wt_	R^2^
Species * RH	10	0	1	0.8842
Species + RH	6	89.24	0	0.8212
Species	4	199.55	0	0.6990
RH	4	432.64	0	0.1145

K = number of parameters in the model including intercept; ΔAIC_c_ = difference in AIC_c_ score between model and most strongly supported model; AIC_wt_ = Akaike weight = probability that model is the most parsimonious among those considered; R^2^ = adjusted proportion of variance explained by model.

**Fig 4:**
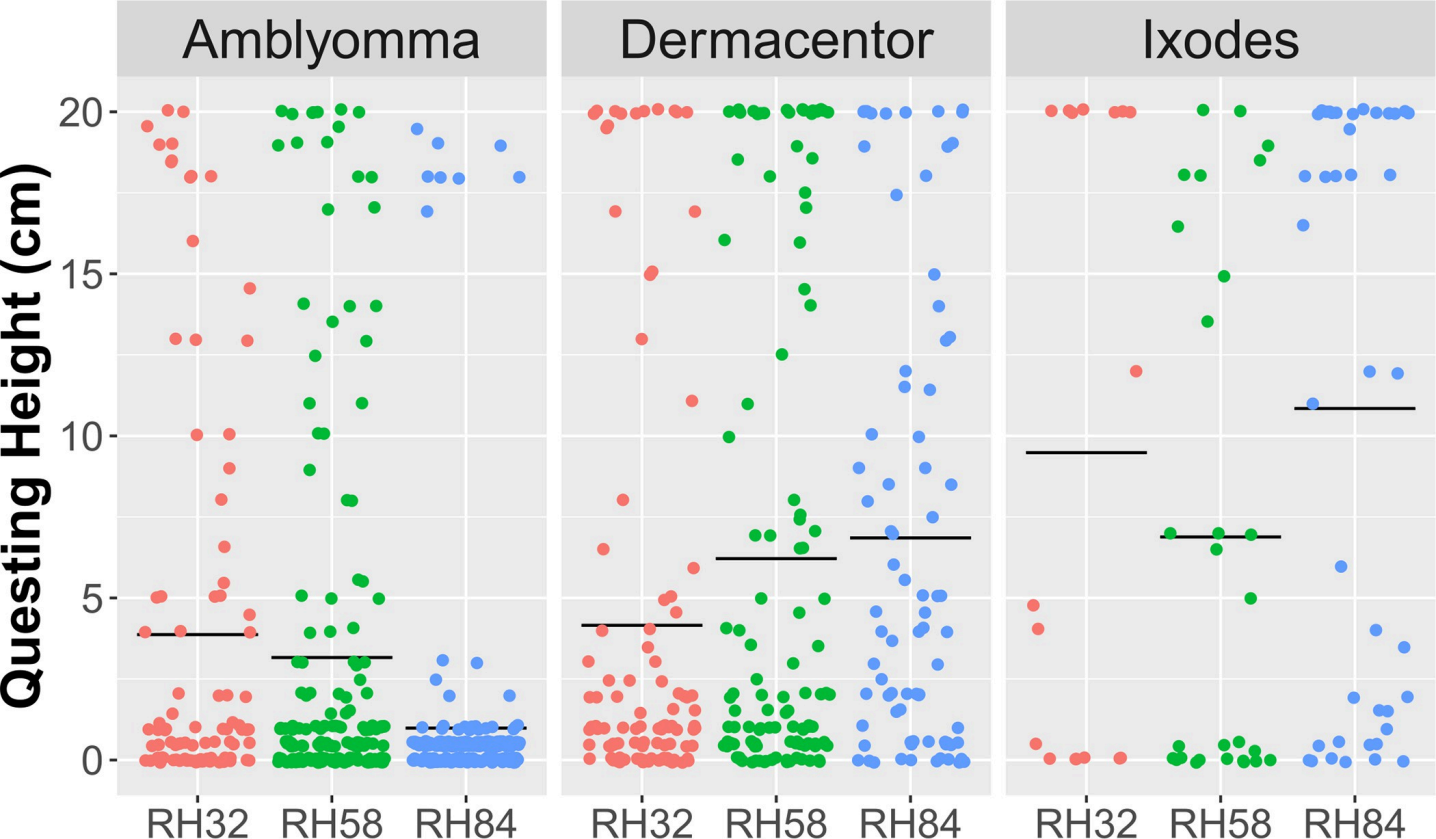
Questing heights of individual ticks in nine experimental groups. Each dot represents a single instance of tick questing; horizontal lines represent the average questing heights of each group. Questing height was negatively associated with RH in *Amblyomma* (H = 24.765, df = 2, P < 0.001), positively associated with RH in *Dermacentor* (H = 11.248, df = 2, P = 0.004), and not associated with RH in *Ixodes* (H = 5.065, df = 2, P = 0.079).

We observed 506 total instances of *Amblyomma* ticks questing at various heights (Fig 4). In a large majority of instances, *Amblyomma* ticks quested at or very near the floor of the tubes. Questing height of *Amblyomma* ticks was negatively associated with RH (P < 0.001). We observed 298 total instances of *Dermacentor* ticks questing. It was common to observe *Dermacentor* ticks questing at the roof of the tubes. Questing height of *Dermacentor* ticks was positively associated with RH (P = 0.004). We observed 86 total instances of *Ixodes* ticks questing. This lower number of questing observations was due to the steep decline in survivorship, such that all *Ixodes* ticks died by day 8 of the experiment (Fig 3). Questing height of *Ixodes* ticks was not associated with RH (P < 0.079).

**Fig 5:**
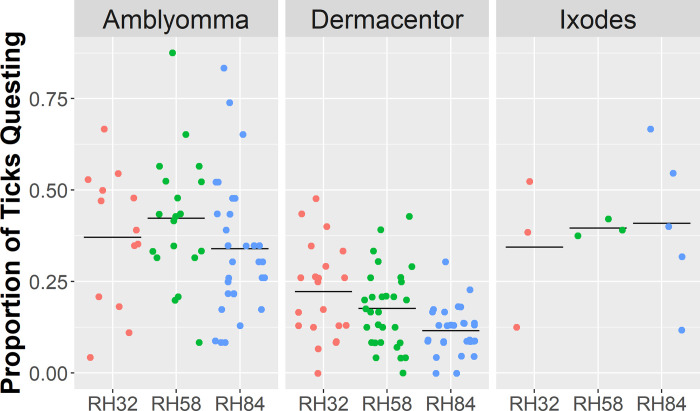
Questing frequencies in nine experimental groups. Each dot represents the proportion of ticks questing out of all living ticks within each experimental group on a particular day; the horizontal lines represent the average questing frequencies of each group. Questing frequency increased with drier conditions in *Dermacentor* (H = 8.792, df = 2, P = 0.012), but not in *Amblyomma* (H = 3.026, df = 2, P = 0.220) or *Ixodes* (H = 0.194, df = 2, P = 0.908).

Questing frequency, the group-level proportion of ticks that were alive and questing on any given day, was generally lower in *Dermacentor* ticks compared to *Amblyomma* and *Ixodes* ticks (Fig 5). However, in within-species comparisons, the questing frequency of *Dermacentor* was highest in the lowest RH (P = 0.012), which represents the most climatically stressful treatment. RH did not affect the questing frequency of *Amblyomma* (P = 0.220) or *Ixodes* (P = 0.908).

## Discussion

We developed a safe, inexpensive, convenient, fast, accurate, and stable protocol that represents an improvement over the classical method of using salt solutions for manipulating RH. The method is thus useful for experiments investigating the effects of climatic stress on ticks. We provide evidence of the protocol’s utility with a pilot experiment on the three most important vectors of tick-borne diseases in North America.

Our experimental results indicate that RH affects the survivorship of *A*. *americanum*, *D*. *variabilis*, and *I*. *scapularis*, with rapid mortality present in the groups exposed to low relative humidity where ticks experience a high vapor pressure deficit. Survival times in our study are consistent with studies that used the classical method of manipulating RH with saturated salt solutions. For example, 50% of *Ixodes scapularis* nymphs died in 10.7 days at 75% RH and 27°C [27]. Although survival times of nymphs in the 1994 study were longer at 85% RH and 27°C, we note three important distinctions with our study. First, the critical equilibrium humidity (CEH) of *Ixodes scapularis* nymphs is 85–89% and that of adults is 89–93% [39]. This means that adult ticks have higher RH requirements before they begin to dehydrate. Our experiment used adult ticks; thus, we expected shorter survival times than published studies that used immature ticks. Second, the traditional method of setting RH with saturated salt solutions can result in an equilibrium RH with variation of ±10% [40]. This means that using a salt like ammonium sulfate to set the RH to 82% can yield humidities up to 92%, well above the CEH of nymphs, thus extending their survival. In our experiment, digital measurements of RH varied ±4% (Fig 2). The highest recorded RH in our 84% RH treatment was 86.1%, still well below the CEH of adults; thus, we expected shorter survival times than published studies that used a more variable method of setting RH. Third, temperature performance curves for myriad animals show that a slight increase in temperature above the optimum can cause a precipitous decline in physiological performance. Our experimental temperature range went up to 30°C; thus, we expected shorter survival times than published studies that used cooler temperatures. Survivorship curves show a consistent pattern of mortality associated with desiccation and with the effects of humidity on *I*. *scapularis* being the most acute. These results are consistent with numerous field studies and indicate that climatic stress, experienced by dry conditions, reduces the survival of ixodid ticks [17–19]. We conclude that humidity is a primary abiotic factor that limits the geographic spread of all three tick species, as maintaining water balance is central to their ability to survive climatically stressful conditions in the face of rising global temperatures [10].

Our results also indicate that RH affects the questing behavior of *A*. *americanum* and *D*. *variabilis*. Specifically, drier conditions caused *Dermacentor* ticks to quest more frequently and at lower heights. This negative relationship between RH and questing height in *Dermacentor* is expected because in nature, migrating vertically in vegetation during the day exposes ticks to desiccating conditions, and questing ticks that are unsuccessful in finding a host descend to the leaf litter to rehydrate [30, 41]. In contrast, drier conditions caused *Amblyomma* ticks to quest at higher heights. The tendency of *Amblyomma* to quest at higher heights in dry conditions was surprising, but may explain why questing *A*. *americanum* are more likely to be collected by drag sampling during mid-day when RH is at its lowest [35, 42]. Since questing height is related to the likelihood of contacting humans and thus, risk of disease transmission to humans [43], these results indicate that climate change may impact transmission rates of *Amblyomma*-vectored diseases differently from *Dermacentor*-vectored diseases. We did not observe significant differences in questing behavior of *I*. *scapularis* across RH treatments. However, previous laboratory experiments showed that both *I*. *scapularis* and *I*. *ricinus* nymphs quested at lower heights in dry conditions [29, 44], and previous field studies showed that dry meteorological conditions curtailed questing activity in *I*. *scapularis* [35, 42]. The lack of evidence of the influence of RH on *Ixodes* questing in this experiment is likely because *Ixodes* ticks experienced 100% mortality by day 8 and there were few observations of *Ixodes* questing. These laboratory and field studies suggest that *I*. *scapularis* is particularly sensitive to climatic stressors, which likely limit the expansion of the Lyme disease vector beyond eastern North America.

### Limitations and broader applications

Our protocol has some limitations but also presents opportunities for future research. The principal limitation is that the experiment was conducted in a highly controlled and artificial environment. This permits us to isolate the effects of RH but limits our ability to investigate how ticks interact with other abiotic and biotic factors, such as wind and the presence of hosts or predators. Field studies would complement insights from laboratory experiments that use this protocol. For example, field observational studies in New York, New Jersey, Rhode Island, and Illinois showed that RH and precipitation affect questing activity and abundance patterns of *A*. *americanum*, *D*. *variabilis*, and *I*. *scapularis* [35, 45–48]. Additional experiments showed that microclimatic conditions affect survival of *Ixodes* ticks, although questing behavior depends largely on geographic origin of populations, suggesting local ecological adaptation [43, 49]. Another limitation may be the possibility of developing mold growth on the wooden skewers, especially at high humidities >80% RH [50]. We did not observe any evidence of mold growth on the skewers or on the ticks during the 30-day experiment, but users of this protocol should inspect all surfaces frequently for mold contamination when working with a high RH.

We developed this protocol specifically to investigate the behavior of individual ticks and maximize replication, but this limits our ability to investigate tick aggregations. For example, newly hatched tick larvae remain in a cluster or “tick bomb” in their natural habitat. Hence, our protocol is best suited for nymph and adult life stages, unless the larval cluster is the unit of analysis. Additionally, the dimensions of the tubes we used limit the ticks to a maximum questing height of 20 cm. Ixodid ticks quest at a wider range of heights in both natural and laboratory settings [41]. Using both longer tubes and taller airtight containers can address this limitation.

Climate change introduces a need for increased understanding of how abiotic conditions affect the habitat suitability of arthropod disease vectors. Our novel method of manipulating the microclimate of individual ticks permits future experiments with temperature and humidity to simulate a wide variety of environmental conditions. Experimentally investigating the impact of temperature alongside humidity on a tick’s ability to survive and engage in host-seeking behaviors will facilitate the creation of better models of tick range expansions. Indeed, climatic suitability models of tick range expansion use correlative approaches to associate environmental variables with the current distribution and characterize what is effectively the species’ realized niche. However, a mechanistic approach that incorporates species-specific biophysical properties to characterize the species’ fundamental niche can improve our ability to predict the dynamics of tick range dynamics [51] and to mitigate the risk of tick-borne diseases. This protocol allows the replicable and repeatable study of the effect of climatic conditions on the viability and behavior of various tick species.

Lastly, the protocol can be easily modified to conduct experiments on the effects of climatic stress on many other small terrestrial arthropods, such as insects, spiders, and mites. The same humidity control packs can be used to manipulate RH with only minor modifications to the physical setup to accommodate animals with different traits, body sizes, or life stages.

## Supporting information

S1 Appendix Step-by-step protocol, also available on protocols.io. (PDF)

S2 Appendix Comparison of cost, preparation time, and performance of humidity control packs and saturated salt solutions. (PDF)
