# Supplementary material for: A novel laboratory method to simulate climatic stress with successful application to experiments with medically relevant ticks
Source: PLoS One. 2022 Sep 29;17(9):e0275314. doi: 10.1371/journal.pone.0275314 (PMC9522300; doi:10.1371/journal.pone.0275314)
Supplement: S1 Appendix — (PDF) [file pone.0275314.s001.pdf]

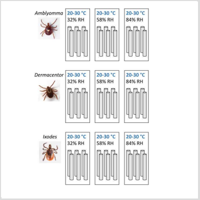

4

May 26, 2022

A novel laboratory method to simulate climatic stress with successful application to experiments with medically relevant ticks V.4

Sang Hyo Kim[1], Caleb Nielebeck[1], Lauren Dedmon[1], Mark Pangilinan[1], Jahred Quan[1], William Ota[1], Javier D. Monzón[1]

[1]Pepperdine University

1

dx.doi.org/10.17504/protocols.io.rm7vzyo8rlx1/v4

Caleb Nielebeck

This protocol details a novel method to isolate individual ticks and manipulate their environment. We successfully used this method to investigate how humidity affects survival and host-seeking (questing) behavior of three species of ticks: the lone star tick (Amblyomma americanum), American dog tick (Dermacentor variabilis), and black-legged tick (Ixodes scapularis). We placed 72 adult females of each species into individual plastic tubes and separated them into three experimental relative humidity (RH) treatments representing distinct climates: 32% RH, 58% RH, and 84% RH. For 30 days we assessed the survival and questing behavior of each tick.

DOI

dx.doi.org/10.17504/protocols.io.rm7vzyo8rlx1/v4

Sang Hyo Kim, Caleb Nielebeck, Lauren Dedmon, Mark Pangilinan, Jahred Quan, William Ota, Javier D. Monzón 2022. A novel laboratory method to simulate climatic stress with successful application to experiments with medically relevant ticks. **protocols.io**
https://dx.doi.org/10.17504/protocols.io.rm7vzyo8rlx1/v4
Caleb Nielebeck

Tick questing, tick behavior experiment, Amblyomma, Dermacentor, Ixodes

protocol ,

May 26, 2022

May 26, 2022

Citation: Sang Hyo Kim, Caleb Nielebeck, Lauren Dedmon, Mark Pangilinan, Jahred Quan, William Ota, Javier D. MonzÃÂ³n A novel laboratory method to simulate climatic stress with successful application to experiments with medically relevant ticks https://dx.doi.org/10.17504/protocols.io.rm7vzyo8rlx1/v4

This is an open access protocol distributed under the terms of the **Creative Commons Attribution License**

63302

Questing qualifications:

- The individual is not walking but it is still with its front legs extended

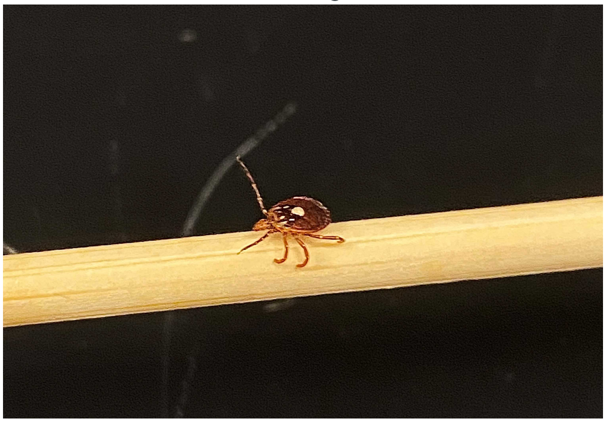

Example of an *Amblyomma* tick questing

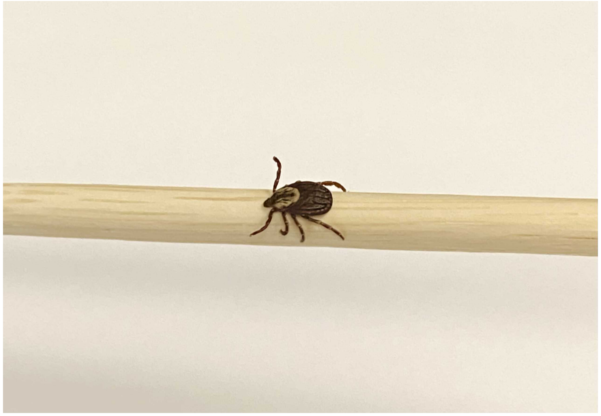

Example of a *Dermacentor* tick questing

Death qualifications:

- If any tick appears dead, lightly blow on it since ticks respond to carbon dioxide exhaled by potential hosts
- If the tick does not move at all in 2 minutes, it should be counted as dead and placed in 70% ethanol

Citation: Sang Hyo Kim, Caleb Nielebeck, Lauren Dedmon, Mark Pangilinan, Jahred Quan, William Ota, Javier D. MonzÃÂ³n A novel laboratory method to simulate climatic stress with successful application to experiments with medically relevant ticks https://dx.doi.org/10.17504/protocols.io.rm7vzyo8rlx1/v4

This is an open access protocol distributed under the terms of the **Creative Commons Attribution License**

Ticks:

- 72 adult female *Amblyomma americanum*
- 72 adult female *Dermacentor variabilis*
- 72 adult female *Ixodes scapularis*

Experimental set up:

- 1 - Climate chamber (e.g. Percival I-41VL)

Incubator
Climate Chamber
Percival **I-41VL**

- 216 - 20 cm x 2.5 cm Clear PETG plastic tubes
- 216 - 20 cm Wooden skewers
- 36 - 2 L Airtight containers
- 12 - **32 %** Boveda Two-Way Humidity Control Packs
- 12 - **58 %** Boveda Two-Way Humidity Control Packs
- 12 - **84 %** Boveda Two-Way Humidity Control Packs
- 1 - Temperature/relative humidity data logger (e.g. ONSET UX100-003)

HOBO Temperature/Relative Humidity 3.5% Data Logger
Data logger
ONSET **UX100-003**

- 70% Ethanol
- Colored dot stickers
- Sharpie

Other tools:

- Entomology forceps
- 30 cm ruler
- White surface (e.g. lab bench diaper)

Always handle ticks with blunt entomology forceps, as regular forceps can injure them.
Always handle ticks over a white surface so that they can easily be spotted in case they are dropped.

Citation: Sang Hyo Kim, Caleb Nielebeck, Lauren Dedmon, Mark Pangilinan, Jahred Quan, William Ota, Javier D. MonzÃÂ³n A novel laboratory method to simulate climatic stress with successful application to experiments with medically relevant ticks https://dx.doi.org/10.17504/protocols.io.rm7vzyo8rlx1/v4

This is an open access protocol distributed under the terms of the **Creative Commons Attribution License**

We acquired adult female ticks from the Oklahoma State University Tick Rearing Facility and experimented with one temperature range and three relative humidities. This protocol can be modified for different species, life stages, temperatures, humidities, and other small organisms.

Set up 2h

1 Place a single tick with one wooden skewer in each tube and seal with a cap, labelling each tube with an individual identifier

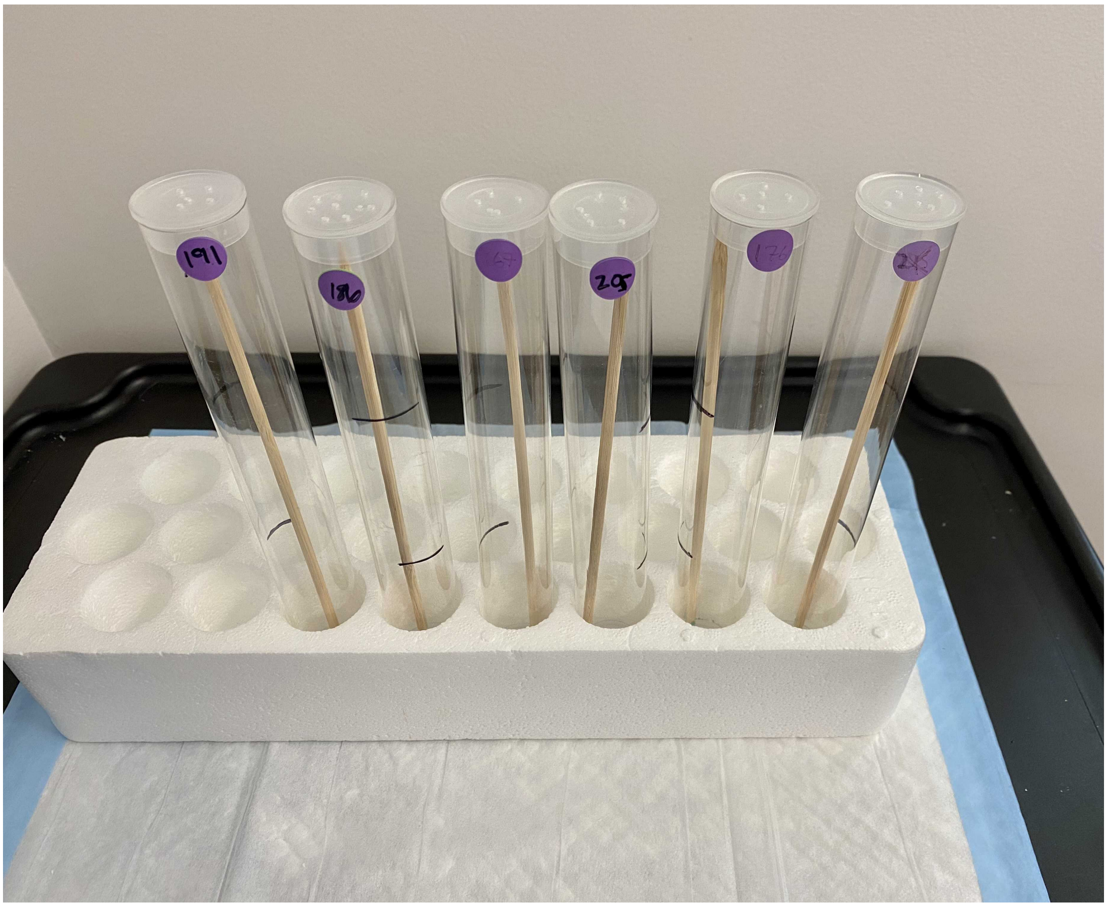

PETG plastic tubes with wooden skewers prepared for ticks. Notice that we drilled holes in the caps to allow airflow between the tubes and the containers.

2 Place six tubes in each airtight container along with a humidity pack, labelling each container

Citation: Sang Hyo Kim, Caleb Nielebeck, Lauren Dedmon, Mark Pangilinan, Jahred Quan, William Ota, Javier D. MonzÃÂ³n A novel laboratory method to simulate climatic stress with successful application to experiments with medically relevant ticks https://dx.doi.org/10.17504/protocols.io.rm7vzyo8rlx1/v4

This is an open access protocol distributed under the terms of the **Creative Commons Attribution License**

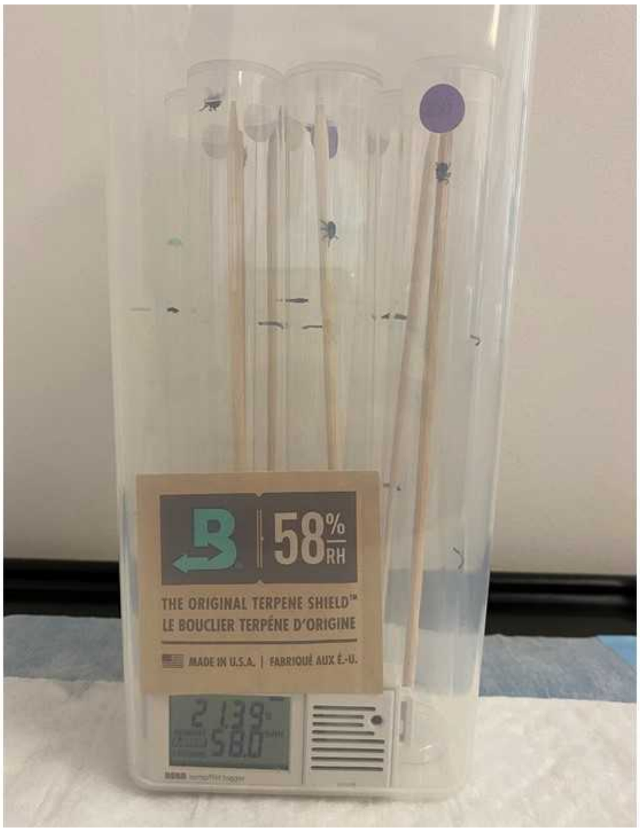

Airtight container with six tubes, humidity pack, and data logger. Example container of *Dermacentor variabilis* (purple) at 58% RH; every container was replicated four times for a sample size of n=24 per RH group.

2.1 Confirm the humidity in one container of each RH level with the data logger

3 Program the climate chamber

Reference your chamber's user's manual

3.1 To cycle between **20 °C** to **30 °C**, the temperature increments should be as follows:

- 3:00 - **25 °C**
- 6:00 - **27.5 °C**
- 9:00 - **30 °C**
- 12:00 - **27.5 °C**

Citation: Sang Hyo Kim, Caleb Nielebeck, Lauren Dedmon, Mark Pangilinan, Jahred Quan, William Ota, Javier D. MonzÃÂ³n A novel laboratory method to simulate climatic stress with successful application to experiments with medically relevant ticks https://dx.doi.org/10.17504/protocols.io.rm7vzyo8rlx1/v4

This is an open access protocol distributed under the terms of the **Creative Commons Attribution License**

- 15:00 - 25 °C
- 18:00 - 22.5 °C
- 21:00 - 20 °C
- 24:00 - 22.5 °C

3.2 To create a 12:12 light:dark photoperiod, lighting increments should be as follows:
- 9:00 - Lights on
- 21:00 - Lights off

Data collection 5w 5d

4 Place all of the airtight containers, filled with ticks and humidity packs, into the climate chamber and start the program

5 Each day thereafter, during the 9:00 to 12:00 or 30 °C increment, assess each tick for survivorship and questing behavior (see guidelines for qualifications of survivorship and questing)

> Only take one container out of the chamber at a time

> Collect a binary outcome for survivorship and questing, and measure the tick's height (to the nearest 0.5 cm) in the tube if it is found questing

5.1 Periodically move the data logger to a new bin to confirm that no unexpected changes to the climate inside the containers has occurred

6 Repeat steps 4 and 5 for 30 days or until all ticks have died

Citation: Sang Hyo Kim, Caleb Nielebeck, Lauren Dedmon, Mark Pangilinan, Jahred Quan, William Ota, Javier D. MonzÃÂ³n A novel laboratory method to simulate climatic stress with successful application to experiments with medically relevant ticks https://dx.doi.org/10.17504/protocols.io.rm7vzyo8rlx1/v4

This is an open access protocol distributed under the terms of the **Creative Commons Attribution License**
