# Supplementary material for: A novel laboratory method to simulate climatic stress with successful application to experiments with medically relevant ticks
Source: PLoS One. 2022 Sep 29;17(9):e0275314. doi: 10.1371/journal.pone.0275314 (PMC9522300; doi:10.1371/journal.pone.0275314)
Supplement: S2 Appendix — (PDF) [file pone.0275314.s002.pdf]

S2 Appendix

A novel laboratory method to simulate climatic stress with successful application to experiments with medically relevant ticks

Caleb Nielebeck[1], Sang Hyo Kim[1], Lauren Dedmon[1], Mark Pangilinan[1], Jahred Quan[1], William Ota[1], Javier D. Monzón[1]*

[1] Natural Science Division, Pepperdine University, Malibu, CA, USA

Table S1. Comparison of cost, preparation time, and performance of three humidity control packs and three saturated salt solutions.

Comparison	RH control pack	Saturated salt solution
Cost per replicate	BOV32: $1.50<br>BOV58: $1.50<br>BOV84: $1.50	$MgCl_2$: $9.27<br>$Ca(NO_3)_2$: $8.83<br>KCl: $1.85
Preparation time per RH treatment	~2 min	~45 min
RH set rate (time to halfway between initial RH and equilibrium RH)	BOV32: 15 min<br>BOV58: 40 min<br>BOV84: 25 min	$MgCl_2$: 43 min<br>$Ca(NO_3)_2$: 129 min<br>KCl: 85 min
Accuracy (target RH - mean RH from 18 to 24 hrs after setup	BOV32: 4.2%<br>BOV58: 4.1%<br>BOV84: 0.2%	$MgCl_2$: 3.9%<br>$Ca(NO_3)_2$: 9.1%<br>KCl: 0.5%
Stability in constant temperature (standard deviation from 18 to 24 hrs after setup)	BOV32: 0.06%<br>BOV58: 0.03%<br>BOV84: 0.09%	$MgCl_2$: 0.07%<br>$Ca(NO_3)_2$: 0.03%<br>KCl: 0.17%
Resilience (time to halfway between disturbed RH and equilibrium RH after opening container for 1 minute at 24 and 27 hours)	BOV32: 28 min, 24 min<br>BOV58: 17 min, 13 min<br>BOV84: 17 min, 13 min	$MgCl_2$: 19 min, 85 min<br>$Ca(NO_3)_2$: 37 min, 11 min<br>KCl: 9 min, 7 min
Stability in cycling temperature (standard deviation from 0 to 24 hrs after equilibrium)	BOV32: 1.10%<br>BOV58: 1.91%<br>BOV84: 0.91%	$MgCl_2$: 0.70%<br>$Ca(NO_3)_2$: 2.05%<br>KCl: 3.03%

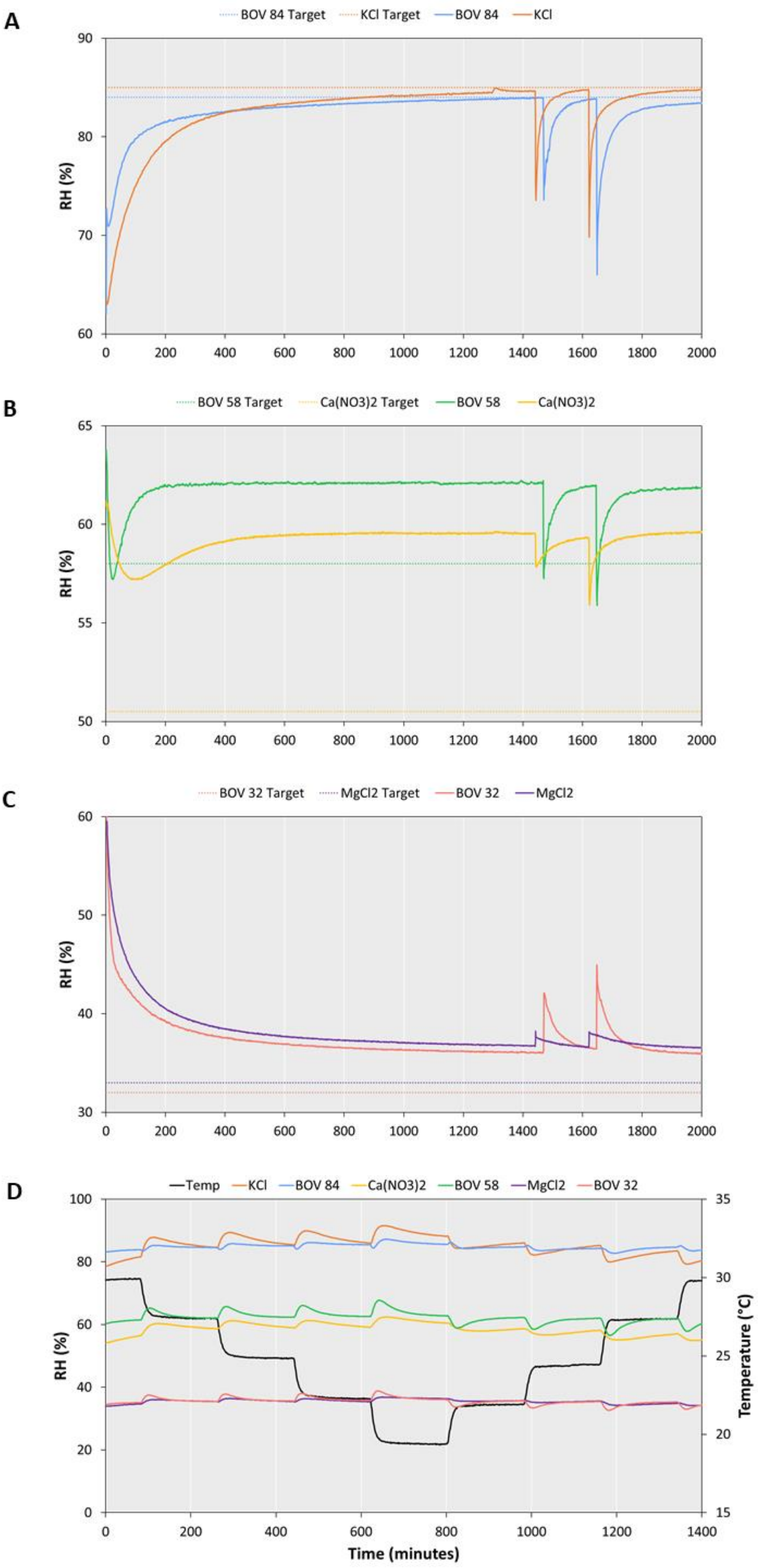
A
BOV 84 Target
KCl Target
BOV 84
KCl
RH (%)
B
BOV 58 Target
Ca(NO3)2 Target
BOV 58
Ca(NO3)2
RH (%)
C
BOV 32 Target
MgCl2 Target
BOV 32
MgCl2
RH (%)
D
Temp
KCl
BOV 84
Ca(NO3)2
BOV 58
MgCl2
BOV 32
RH (%)
Temperature (°C)
Time (minutes)

Figure S1. Performance of three humidity control packs and three saturated salt solutions in a constant temperature of 25 °C (A-C) and in a cycling temperature of 20-30 °C (D). HOBO data loggers recorded temperature and RH each minute. The constant temperature trial (A-C) started at ambient room RH (~60%) and 25 °C; the airtight containers were opened for 1 minute at 24 and 27 hours to disturb the equilibrium RH. The cycling temperature trial (D) started at equilibrium RH and 30 °C.
